# Behavior Change Interventions Delivered through Interpersonal Communication, Agricultural Activities, Community Mobilization, and Mass Media Increase Complementary Feeding Practices and Reduce Child Stunting in Ethiopia

**DOI:** 10.1093/jn/nxz087

**Published:** 2019-06-05

**Authors:** Sunny S Kim, Phuong Hong Nguyen, Yisehac Yohannes, Yewelsew Abebe, Manisha Tharaney, Elizabeth Drummond, Edward A Frongillo, Marie T Ruel, Purnima Menon

**Affiliations:** 1 Poverty, Health, and Nutrition Division, International Food Policy Research Institute, Washington, DC; 2 Alive & Thrive, FHI 360, Addis Ababa, Ethiopia; 3 Alive & Thrive, FHI 360, Washington, DC; 4 Save the Children USA, Washington, DC; 5 Arnold School of Public Health, University of South Carolina, Columbia, SC; 6 Poverty, Health, and Nutrition Division, International Food Policy Research Institute, New Delhi, India

**Keywords:** Ethiopia, child undernutrition, complementary feeding, infant feeding, young child feeding, effectiveness evaluation

## Abstract

**Background:**

Appropriate infant and young child feeding practices are critical for optimal child growth and development, but in Ethiopia, complementary feeding (CF) practices are very poor. Alive & Thrive (A&T) provided intensive behavior change interventions through 4 platforms: interpersonal communication (IPC), nutrition-sensitive agricultural activities (AG), community mobilization (CM), and mass media (MM).

**Objectives:**

The aim of this study was to evaluate the impact of A&T intensive compared with nonintensive interventions (standard nutrition counseling and agricultural extension service and less intensive CM and MM) on CF practices and knowledge and child anthropometric outcomes.

**Methods:**

We used a cluster-randomized evaluation design with cross-sectional surveys among households with children aged 6–23.9 mo [*n *= 2646 at baseline (2015) and *n *= 2720 at endline (2017)]. We derived difference-in-difference impact estimates (DDEs) and conducted dose–response and path analyses to document plausibility of impacts.

**Results:**

At endline, exposure to IPC was 17.8–32.3%, exposure to AG was 22.7–36.0%, exposure to CM was 18.6–54.3%, and exposure to MM was 35.4% in the intensive group. Minimum dietary diversity and minimum acceptable diet increased significantly in the intensive group but remained low at endline (24.9% and 18.2%, respectively). Significant differential declines in stunting prevalence were observed (DDE: −5.6 percentage points; *P* < 0.05) in children aged 6–23.9 mo, decreasing from 36.3% to 22.8% in the intensive group. Dose–response analyses showed higher odds of minimum dietary diversity (OR: 3.3; 95% CI: 2.2, 4.8) and minimum meal frequency (OR: 1.9; 95% CI: 1.4, 2.6) and higher height-for-age *z* score (HAZ) (β: 0.24; 95% CI: 0.04, 0.4) among women exposed to 3 or 4 platforms. Path analyses showed a strong relation between AG and egg consumption, which led to increased child dietary diversity and HAZ.

**Conclusions:**

Delivery of social and behavior change interventions using multiple platforms was feasible and effective, resulting in improvements in CF practices and child stunting within a 2-y period. There is a need for continued efforts, however, to expand intervention coverage and to improve CF practices in Ethiopia. This trial was registered at clinicaltrials.gov as NCT02775552.

## Introduction

Ethiopia has made considerable progress in reducing infant, child, and maternal mortality during the past decade by expanding primary health care services and improving the quality of health service provision (1). Child undernutrition remains high, however, with a prevalence of stunting of 38% in children <5 y of age in 2016, down from 44% in 2011. Underweight affects 24% of children <5 y old, and wasting affects 10% of children and its prevalence remained unchanged between 2011 and 2016 (2).

Appropriate infant and young child feeding (IYCF) practices, which include exclusive breastfeeding (EBF) until 6 mo of age and the age-appropriate provision of safe and nutritious foods in sufficient quantity, in addition to breast milk, from 6 to 23 mo of age, are important for optimal child growth and development (3–7). In Ethiopia, breastfeeding (BF) is universal and practiced for a relatively long period, with 92% continued BF at age 1 y and 76% at age 2 y, although EBF of children <6 mo of age was 58% in 2016, up from 52% in 2011 (2). Complementary feeding (CF) practices, however, are very poor, with 14% minimum dietary diversity and 45% minimum meal frequency for children aged 6–23 mo. Thus, effective strategies to improve CF practices are critical.

The evidence base on impacts of different combinations of interventions to achieve these recommended practices is growing (3, 4, 8–12). Recent impact evaluations of large-scale social and behavior change communication interventions to improve IYCF practices in several countries have shown that intensive interpersonal counseling combined with mass media (e.g., television spots and radio campaigns) and community mobilization activities (e.g., community group meetings, cooking demonstrations, and theater/video shows) have positive impacts on BF (13, 14) and CF practices (13, 15, 16). In Bangladesh, combined intensive interventions resulted in significant improvements in EBF [36 percentage points (pp)], early initiation of BF (17 pp), minimum dietary diversity (16 pp), minimum meal frequency (15 pp), minimum acceptable diet (22 pp), and consumption of iron-rich foods (25 pp) (14, 15). Similar results were observed in Vietnam (16).

In Ethiopia, increased EBF, early initiation of BF, and multiple CF practices were observed in intervention areas (13), although low exposure to interventions, primarily delivered through the government health system, was identified as a challenge (17). A community-based participatory nutrition promotion intervention delivered by a nongovernmental organization in addition to existing government nutrition programs, compared with the government programs alone, showed decreased prevalence of stunting and underweight among children in the intervention areas compared with the control group (18). Thus, evidence of effective complementary behavior change interventions and of higher intensity of exposure to interventions associated with IYCF practices (13) points to a need to reinforce delivery systems and/or to leverage multiple platforms for nutrition interventions.

Linking nutrition with agriculture by making agricultural programs nutrition-sensitive (e.g., by improving targeting and strengthening nutrition-focused actions) has been suggested to reinforce the reach of nutrition-specific interventions and to create enabling conditions for children to grow and develop (19, 20). An evaluation of an integrated agriculture and nutrition and health behavior change communication program in Burkina Faso showed impacts on anemia and wasting among children aged 3–12 mo, as well as multiple IYCF practices (21). A 12-mo integrated intervention package of agricultural inputs and training with nutrition and health education in Ghana had impacts on children's minimum dietary diversity and positive effects on growth (22). Integrated approaches or interventions involving the agricultural sector, therefore, have the potential to enhance nutritional outcomes.

Given the evidence of poor CF practices in Ethiopia and that combined or integrated interventions work, Alive & Thrive (A&T) leveraged multiple platforms to deliver IYCF-related social and behavior change interventions. This article reports on findings from a cluster-randomized impact evaluation of A&T interventions. We hypothesized that the A&T intervention package would have positive impacts on CF practices and knowledge and anthropometric outcomes among children aged 6–23.9 mo.

## Methods

### Program description

A&T is an initiative to save lives, prevent illness, and contribute to healthy growth and development through improving IYCF practices. In phase I (2009–2014), A&T operated in Bangladesh, Ethiopia, and Vietnam, reaching millions of children <2 y old through large-scale social and behavior change communication interventions and achieving substantial gains in IYCF practices (13–16). The focus of phase II (2015–2017) in Ethiopia was to operationalize the Government of Ethiopia's National Nutrition Plan in one region, Amhara, to improve IYCF practices using a multisectoral approach.

In 3 western zones of Amhara, A&T with Save the Children as its implementing partner worked with government health extension workers (HEWs), health development team leaders (HDTLs; a cadre of community health volunteers), and agricultural extension workers to deliver IYCF messages through interpersonal communication (IPC) and promote nutrition-sensitive agricultural activities (AG) to benefit children <2 y old. In intensive intervention areas, HEWs provided IYCF-focused counseling during health post visits and home visits and conducted food demonstrations, HDTLs provided IYCF-focused messaging during home visits, and agricultural extension workers promoted AG activities such as designating a chicken whose eggs are prioritized for a child <2 y old in the household and prioritizing vegetables from home gardens for those children (no inputs were provided as part of the program). The Ethiopian Orthodox Church priests and leaders delivered community mobilization (CM) activities such as sermons about adequate child feeding during religious fasting periods, which are common and extensive in the region, and enhanced community conversations about IYCF were led by community-based organizations. In nonintensive areas, HEWs and HDTLs provided standard nutrition counseling and food demonstrations as feasible, without additional implementation support from A&T; agricultural extension workers provided standard agricultural services; and little or no IYCF-focused CM activities were held. However, there was some spillover of activities, as A&T tools and materials were being adopted by government, nongovernmental organizations, and other stakeholders in the country. The mass media (MM) component, implemented in both intensive and nonintensive areas, consisted of a regional broadcast of radio drama called “Sebat Mela” (translated as “Seven Wisdoms”), which included 12 episodes with stories that aligned with A&T's IYCF messages, associated jingles, and testimonials of model mothers. In intensive areas with limited access to radio, supplemental activities were conducted, including broadcasting the radio drama through mobile vans with speakers and utilizing traveling performers to enact parts of the drama. Thus, A&T used 4 different platforms—that is, IPC, AG, CM, and MM—to deliver behavior change interventions to targeted beneficiaries. The intensive group received all the interventions; the nonintensive group received standard IPC and AG and less intensive CM and MM. These platforms and the specific interventions were developed based on the experiences and lessons from A&T phase I in Ethiopia.

### Evaluation design

We used a cluster-randomized, nonblinded impact evaluation design with repeated cross-sectional surveys to assess the impact of the A&T intensive intervention package compared to a nonintensive program. A cross-sectional household survey was conducted at baseline (2015) and exactly 2 years later (2017) in the same communities in households with children <2 y old. This article presents findings on the primary outcomes for the evaluation (i.e., the WHO-recommended core CF practices) and the secondary outcomes of maternal knowledge about CF and stunting prevalence among children aged 6–23.9 mo.

### Sample size estimations

Sample size was calculated to detect differences in the primary outcomes of CF practices for children aged 6–23.9 mo, between the 2 intervention groups, considering an α of 0.05, a power of 0.80, and an intraclass correlation of 0.02, and estimated baseline prevalence of the primary outcomes. Assuming a baseline prevalence of 5.1% for minimum dietary diversity, we estimated that a total sample of 2700 children aged 6–23.9 mo (1350/group) was sufficient to detect a minimum of 6-pp difference in the proportion of children achieving minimum dietary diversity. With a baseline prevalence of 55.8% for minimum meal frequency, this total sample size was also sufficient to detect a minimum of 10-pp difference in the proportion of children achieving minimum meal frequency.

### Random assignment and blinding

A cluster was defined as a rural *woreda* (district). Among the total of 41 woredas in the 3 western zones of Amhara, Save the Children selected 20 woredas as possible A&T intensive areas on the basis of being first- or second-level agriculturally productive areas, not participating in the Productive Safety Net Program (a national cash and food transfer program targeted to chronically food insecure households), geographic proximity, size, and other operational aspects to ensure homogeneity across the sample. We stratified randomization by zone, and the woredas were randomly assigned to either the intensive (10 woredas) or the nonintensive (10 woredas) intervention by use of computer-generated pseudo-random numbers. All communities within an allocated woreda received the same intensive or nonintensive interventions. Households in the intensive and nonintensive areas were not explicitly informed about the results of the randomization. There was no blinding of the intervention at the level of service delivery.

### Outcomes

The primary outcomes were CF practices in children aged 6–23.9 mo, and the secondary outcomes were maternal CF knowledge and prevalence of stunting among children aged 6–23.9 mo. CF practices were measured based on the indicators recommended by WHO (23). Five CF indicators were examined: *1*) minimum dietary diversity (defined as the consumption of foods from ≥4 of 7 food groups in the previous 24 h); *2*) minimum meal frequency (defined as the frequency of consuming foods as appropriate for age and BF status); *3*) minimum acceptable diet (defined as BF, achievement of the minimum dietary diversity, and age-appropriate minimum meal frequency); *4*) consumption of iron-rich or iron-fortified foods; and *5*) timely introduction of solid, semisolid, or soft foods (24). The CF indicators were constructed based on maternal previous 24-h recall of foods consumed. For the total number of food groups consumed, all liquids and foods consumed by the child during the previous day were classified into 7 food groups based on a standardized method (24).

Maternal CF knowledge was assessed based on mothers’ responses to a set of 12 questions about CF. Items were validated in a previous study (25) and adapted for our study context. Each knowledge item was given a score of 1 (correct) or 0 (incorrect), and the sum was used as the CF knowledge scores (scale: 0–12).

Anthropometric data were collected by using a standardized method (26). Locally manufactured collapsible length boards, which were precise to 1 mm, were used to measure the recumbent length of children. Weights of the children were measured using electronic weighing scales that were precise to 100 g. Weight and length were converted into height-for-age *z* scores (HAZs), weight-for-age *z* scores (WAZs), and weight-for-height *z* scores (WHZs), according to the WHO child growth standards (27). Stunting, underweight, and wasting were defined as <−2 SD for HAZ, WAZ, and WHZ, respectively.

### Intervention exposure measures

Exposure to the 4 different intervention platforms described previously (i.e., IPC, AG, CM, and MM) was defined by measures of exposure to individual interventions as follows: IPC exposure measured by mothers’ report of receiving IYCF messages during HEW home visit, health post visit, or HDTL home visit in the last 3 months; AG exposure measured by receipt of messages about raising a “baby's chicken” or a “baby's vegetable garden”; CM exposure measured by participation in a food demonstration, enhanced community conversation, or a priest sermon about child feeding and fasting; and MM exposure measured by hearing of the Sebat Mela radio program. Exposure to multiple platforms was defined as any combination of platforms exposed, with a range of 0–4 platforms.

### Statistical analysis

Differences in sample characteristics at baseline and endline between the 2 intervention groups were tested using linear regression models (for continuous variables) or logit regression models (for categorical variables), accounting for geographic clustering (28). For analyses of impact, we derived difference-in-difference impact estimates (DDEs) using fixed-effects regression models that assessed differences between the intensive and nonintensive groups over time (29). We present pure intention-to-treat DDEs; adjusted DDEs that control for geographical clustering and child age and sex; and models fully adjusted for geographic clustering, child age and sex, baseline characteristics that were different between groups, and characteristics that changed differentially over time. Dose–response analyses were conducted using regression models with exposure variables constructed from individual interventions and 4 categories of platforms (IPC, AG, CM, and MM). We also conducted path analyses to examine the linkage between intervention exposure to child HAZ through minimum dietary diversity. To test the accuracy of self-reported outcome measures, we measured social desirability to assess and account for potential bias in our main impact estimates on CF practices. Social desirability, the tendency of respondents to act in a manner that is viewed favorably by others, was measured with the use of a scale based on a subset of 5 items adapted from Reynolds’ short forms of the Marlowe–Crowne social desirability scale (30). Data analysis was performed using Stata 15 (Stata Corporation).

### Ethical approval

Approval for the study was obtained from the institutional review boards of the Ministry of Science and Technology in Ethiopia and the International Food Policy Research Institute. All mothers of study children were provided with detailed information about the study at recruitment. Verbal informed consent was obtained from mothers prior to their participation in the survey.

## Results

### Trial flow

**FIGURE 1:**
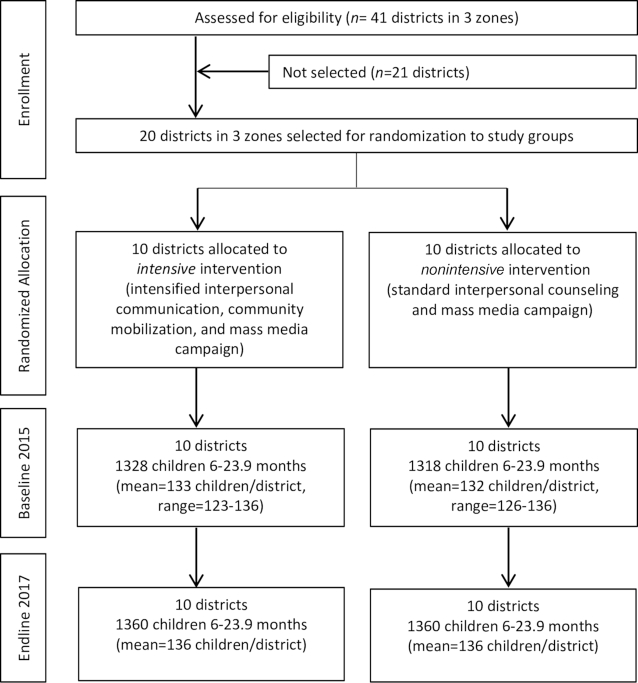
Trial profile.

There were no evaluation clusters lost to follow-up at endline, and there was little variation in cluster size over time (**Figure 1**).

### Sample characteristics

The baseline and endline characteristics of households with children aged 6–23.9 mo by intervention group are shown in **Table 1**. Based on the baseline characteristics, we observed that the random assignment was successful and resulted in a well-balanced set of key characteristics that might be related to intervention uptake or effectiveness. Although we observed no significant differences in key characteristics between intervention groups at endline, household hygiene score, mothers’ education and occupation, number of antenatal care visits, delivery at a health facility, and child age changed over time but with no differential change between groups.

**TABLE 1:** Characteristics of study samples by survey round^1^

	Baseline 2015 (T_1_)		Endline 2017 (T_2_)		Intensive T_2_ – T_1_, pp/mean difference	Nonintensive T_2_ – T_1_, pp/mean difference	* P* ^2^
Characteristics	Intensive (*n *= 1328)	Nonintensive (*n *= 1318)	Intensive (*n *= 1360)	Nonintensive (*n *= 1360)			
Household factors							
Religion: Orthodox Christian, %	98.19	93.92	97.57	94.78	−0.62	0.86	0.31
Children aged <5 y, *n*	1.38 ± 0.53	1.35 ± 0.51	1.35 ± 0.54	1.33 ± 0.53	−0.03	−0.2	0.81
Ownership of house, %	83.06	77.31	82.72	76.10	−0.34	−1.21	0.82
Ownership of garden, %	15.40	15.07	16.99	11.91	1.59	−3.16	0.15
Ownership of agricultural land, %	66.27	65.25	67.43	67.06	1.16	1.81	0.84
SES index,^3^*n*	−0.00 ± 0.83	−0.08 ± 0.89	0.06 ± 0.84	−0.02 ± 0.85	0.07	0.06	0.92
Food security score (range: 0–27), *n*	3.00 ± 4.73	2.86 ± 4.61	3.08 ± 4.94	2.50 ± 4.42	0.08	−0.36	0.32
Food insecurity, %	42.10	41.63	42.50	37.21	0.40	−4.42	0.33
Household dietary diversity (range: 0–12), *n*	6.20 ± 1.32	6.29 ± 1.27	6.36 ± 1.32	6.17 ± 1.39	0.16	−0.13	0.10
Household hygiene score (range: 0–10), *n*	5.51 + 3.50	5.97 + 3.32	5.84 + 3.47	5.72 + 3.62	0.32*	−0.25	0.19
Maternal factors							
Age, y	28.26 ± 6.11	28.09 ± 6.12	28.51 ± 6.09	28.51 ± 5.95	0.24	0.42	0.61
Education (range: 0–16), y	1.58 ± 3.21	1.97 ± 3.57	2.37 ± 3.68	2.55 ± 3.85	0.78*	0.58***	0.51
Occupation as housewife, %	85.39	82.60	72.57	76.76	−12.82**	5.83*	0.05
BMI, kg/m^2^	19.80 ± 2.36	19.94 ± 2.44	20.09 ± 2.35	20.16 ± 2.45	0.29**	0.23*	0.57
Maternal dietary diversity (range: 0–10), *n*	2.75 ± 0.83	2.73 ± 0.83	2.80 ± 0.96	2.75 ± 1.03	0.05	0.02	0.75
Health services access							
Antenatal care visits, *n*	3.84 ± 1.70	3.83 ± 1.71	4.09 ± 1.36	3.96 ± 1.36	0.25**	0.13	0.24
Delivered birth at health facility, %	48.39	55.75	73.88	74.91	25.48***	19.15***	0.10
Children received full immunization, %	48.72	46.74	46.10	46.25	−2.62	−0.49	0.59
Child factors							
Sex (boys), %	51.96	49.54	49.19	49.63	−2.77	0.09	0.50
Age, mo	14.25 ± 5.02	14.37 ± 5.24	13.71 ± 5.11	13.61 ± 5.14	−0.54*	−0.76*	0.49
Acute respiratory infection,^4^ %	9.67	8.91	9.78	10.00	0.11	1.09	0.61
Diarrhea,^4^ %	21.10	17.90	22.13	20.66	1.03	2.76	0.28

1 Values are percentages or means ± SDs unless otherwise indicated. *, **, ***Significant change from baseline to endline:*P < 0.05, **P < 0.01, ***P < 0.001. pp, percentage point; SES, socioeconomic status; T, time.

2 Significant difference between the changes in intensive compared with nonintensive areas, adjusted for geographic clustering effect at woreda level.

3 SES index was constructed by using principal components analysis with variables on ownership of assets; it is a standardized score with mean = 0 and SD = 1.

4 Acute respiratory infection and diarrhea were measured through maternal recall of symptoms in the 2 wk before the survey.

### Impact on IYCF practices

**FIGURE 2:**
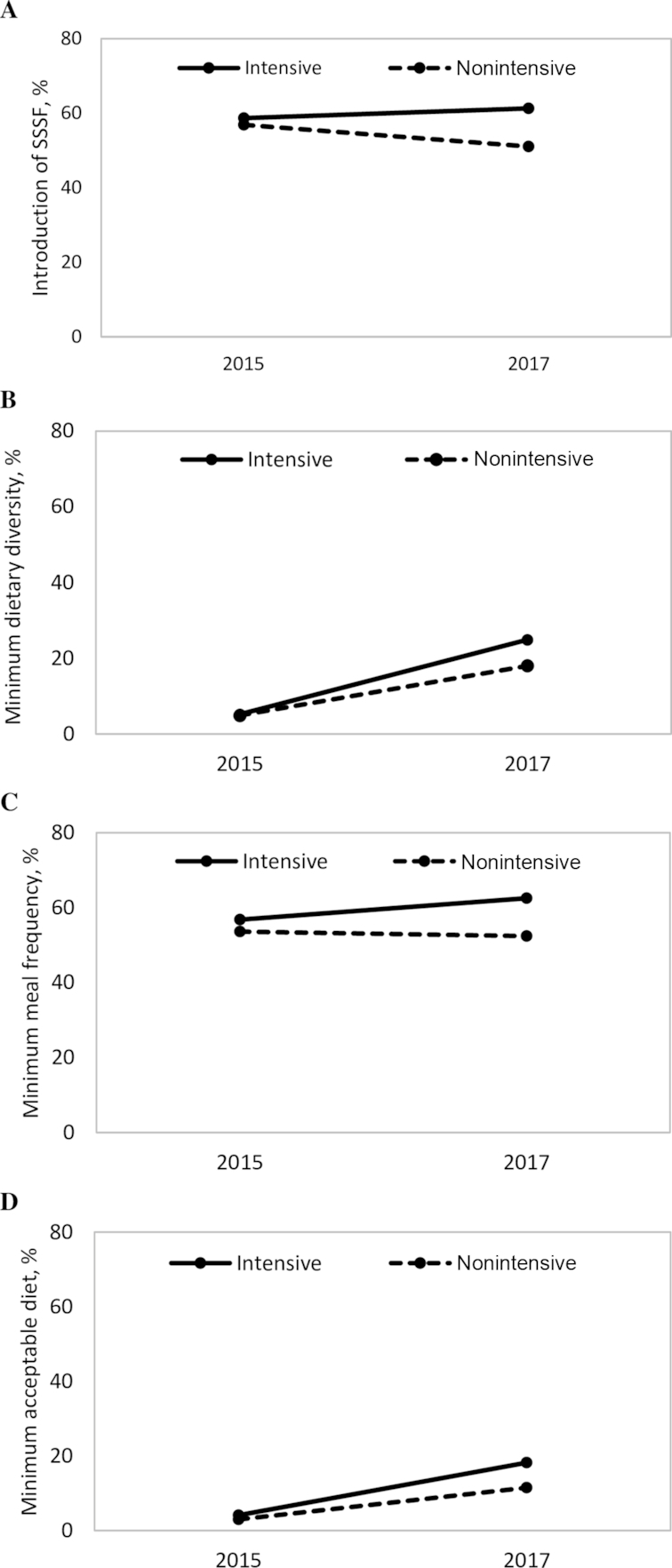
Complementary feeding practices in children aged 6–23.9 mo by intervention group and survey round. (A) Introduction of SSSF, (B) minimum dietary diversity, (C) minimum meal frequency, and (D) minimum acceptable diet. Values are percentages. SSSF, solid, semisolid, or soft foods.

Among all core WHO CF indicators (**Supplemental Table 1**), levels of minimum dietary diversity and minimum acceptable diet improved significantly over time (*P* < 0.001) in both intensive and nonintensive groups, but the increases were marginally higher in the intensive group (*P *= 0.08 and *P *= 0.07, respectively) (**Figure 2**). The DDEs of program impact were 6.6 pp and 5.5 pp for minimum dietary diversity and minimum acceptable diet, respectively. In the intensive areas, minimum dietary diversity increased from 5.2% to 24.9% between baseline and endline, and minimum acceptable diet increased from 4.1% to 18.2%. Minimum meal frequency increased from 56.8% to 62.5% over time in the intensive areas, whereas levels decreased in nonintensive areas, but the differential impact was not statistically significant (*P *= 0.22).

We assessed the individual food groups, which make up the minimum dietary diversity indicator, consumed by children during the previous 24-h period (**Table 2**). In both intensive and nonintensive groups, consumption of eggs, vitamin A-rich fruits and vegetables, and other fruits and vegetables increased significantly over time. There were statistically significant differential improvements in the intensive areas for vitamin A-rich fruits and vegetables only (9.0 pp), which include carrots and kale and other green leafy vegetables—food items specifically promoted by the program through messages and during food demonstrations. This impact remained significant in the fully adjusted impact models.

**TABLE 2:** Reported intake of food groups in the previous 24 h among children aged 6–23.9 mo by intervention group and survey round^1^

	Baseline 2015 (T_1_)		Endline 2017 (T_2_)		Intensive T_2_ – T_1_, pp/mean difference	Nonintensive T_2_ – T_1_, pp/mean difference	Pure ITT DDE,^2^ pp/mean difference (95% CI)	Adjusted ITT DDE,^3^ pp/mean difference (95% CI)	Fully adjusted DDE,^4^ pp/mean difference (95% CI)
Indicator	Intensive (*n *= 1328)	Nonintensive (*n *= 1318)	Intensive (*n *= 1360)	Nonintensive (*n *= 1360)					
Food groups consumed, %									
Grains, roots, and tubers	87.58	87.41	91.62^##^	86.91	4.04	−0.49	4.54 (−0.95, 10.02)	4.07 (−0.87, 9.02)	4.04 (−0.99, 9.07)
Legumes and nuts	57.68	53.87	58.75	55.59	1.07	1.72	−0.65 (−14.70, 13.40)	−1.26 (−14.82, 12.30)	−1.07 (−14.38, 12.24)
Dairy	16.87	18.74	19.63	19.85	2.76	1.11	1.65 (−4.61, 7.91)	1.64 (−4.61, 7.90)	1.35 (−4.93, 7.63)
Flesh foods	2.26	2.20	2.72	3.16	0.46	0.96	−0.50 (−2.09, 1.09)	−0.53 (−2.14, 1.08)	−0.68 (−2.36, 0.99)
Eggs	8.73	7.81	18.46	14.49	9.72**	6.67**	3.05 (−2.61, 8.71)	3.00 (−2.63, 8.63)	2.94 (−2.71, 8.59)
Vitamin A-rich fruits and vegetables	9.26	10.09	21.18^#^	13.01	11.91**	2.92	8.99* (2.45, 15.53)	8.85* (2.34, 15.36)	8.74* (2.18, 15.31)
Other fruits and vegetables	1.43	1.67	44.93	39.71	43.50***	38.04***	5.46 (−1.94, 12.86)	5.23 (−1.93, 12.38)	4.99 (−1.91, 11.89)
Any animal source foods (dairy, flesh food, or eggs), %	23.72	24.66	31.03	29.71	7.31*	5.05	2.26 (−6.32, 10.85)	2.21 (−6.35, 10.78)	1.95 (−6.70, 10.60)
No. of food groups, *n*	1.84 ± 1.01	1.82 ± 1.02	2.57 ± 1.45^#^	2.33 ± 1.40	0.73***	0.51**	0.23 (−0.10, 0.55)	0.21 (−0.10, 0.52)	0.20 (−0.10, 0.51)

1 Values are percentages or means ± SDs unless otherwise indicated. *, **, ***Significant change from baseline to endline in intensive and nonintensive areas separately, adjusted for clustering effect at woreda level: *P < 0.05, **P < 0.01, ***P < 0.001.^#^^, ##^Significant change between A&T intensive and nonintensive areas in the same survey round, adjusted for clustering effect at woreda level: ^#^*P* < 0.05, ^##^*P* < 0.01. DDEs with clustered SEs compare A&T intensive and nonintensive areas in 2015 and 2017. A&T, Alive & Thrive; DDE, difference-in-difference estimate; ITT, intent-to-treat; pp, percentage point; T, time.

2 Accounts for geographic clustering effect at woreda level only.

3 Accounts for geographic clustering effect, child sex, and child age.

4 Accounts for geographic clustering effect, child sex, child age, and variables that are different at baseline and endline (mother's occupation, institutional delivery, and household dietary diversity).

We found no evidence of social desirability bias for any of the CF practices. There was no significant difference in social desirability scores between intensive and nonintensive groups (**Supplemental Table 2**), and no significant difference in reported CF indicators was associated with higher social desirability scores in either group (**Supplemental Table 3**).

### Impact on maternal CF knowledge

**FIGURE 3:**
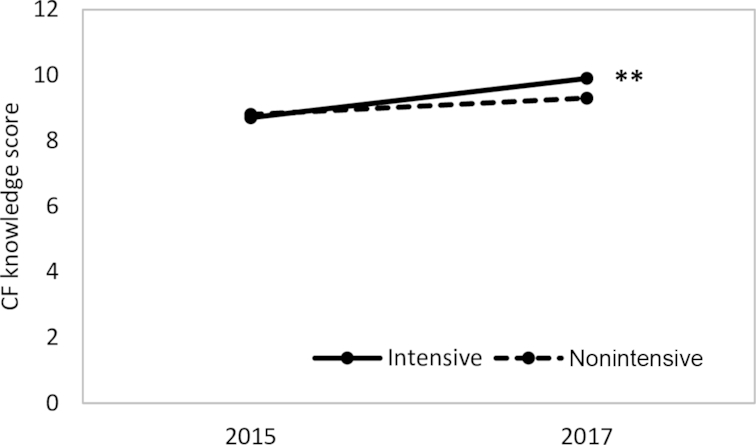
Complementary feeding knowledge scores among mothers with children aged 6–23.9 mo by intervention group and survey round. Values are knowledge score points. **Significant change between groups from baseline to endline, *P* < 0.01. DDEs with clustered SEs comparing Alive & Thrive intensive and nonintensive areas in 2015 and 2017. Accounts for geographic clustering effect at woreda level. CF, complementary feeding; DDE, difference-in-difference estimate.

We also observed a significant impact on maternal CF knowledge scores. The DDE of program impact was 0.7 points for the overall score (**Figure 3**). Among the individual knowledge items, the largest improvement was in the knowledge about special foods such as milk, egg, carrots, and green leafy vegetables for enriching the child's porridge (DDE: 14.6 points), which corroborates the results on dietary diversity and the consumption of vitamin A-rich fruits and vegetables (**Supplemental Table 4**).

### Impacts on stunting and other anthropometric indicators

Stunting declined significantly among children aged 6–23.9 mo in both groups between baseline and endline, with significant differential improvement in favor of the intensive group (DDE: −5.6 pp) in both the pure intention-to-treat and fully adjusted models (**Table 3**). Underweight and wasting also decreased in both groups over time, but the declines in the prevalence did not differ between groups. Improvements in mean HAZ, WAZ, or WHZ did not differ between groups.

**TABLE 3:** Anthropometric indicators among children 6–23.9 mo by intervention group and survey round^1^

	Baseline 2015 (T_1_)		Endline 2017 (T_2_)		Intensive T_2_ – T_1_, pp/mean difference	Nonintensive T_2 –_ T_1_, pp/mean difference	Pure ITT DDE,^2^ pp/mean difference (95% CI)	Adjusted ITT DDE,^3^ pp/mean difference (95% CI)	Fully adjusted DDE,^4^ pp/mean difference (95% CI)
Indicator	Intensive (*n *= 1328)	Nonintensive (*n *= 1318)	Intensive (*n *= 1360)	Nonintensive (*n *= 1360)					
Stunting, *%*	36.27	35.42	22.78	27.58	−13.48***	−7.85**	−5.6* (−11.1, −0.2)	−5.9* (−11.6, −0.3)	−6.0* (−11.7, −0.2)
6–11.9 mo	27.01	26.18	14.07	17.61	−12.93***	−8.57**	−4.4 (−11.5, 2.7)	−4.2 (−11.4, 2.9)	−4.2 (−11.3, 2.8)
12–17.9 mo	37.58	41.08	23.23	30.70	−14.35**	−10.38*	−4.0 (−15.7, 7.7)	−3.7 (−14.8, 7.3)	−3.9 (−14.9, 7.2)
18–23.9 mo	48.01	41.54	34.88	38.50	−13.13*	−3.04	−10.1 (−20.7, 0.5)	−10.2 (−20.8, 3.3)	−10.0 (−20.8, 0.7)
HAZ	−1.33 ± 1.60	−1.34 ± 1.51	−1.05 ± 1.41	−1.17 ± 1.44	0.28**	0.18**	0.10 (−0.10, 0.30)	0.12 (−0.09, 0.33)	0.12 (−0.09, 0.33)
6–11.9 mo	−0.71 ± 1.77	−0.83 ± 1.65	−0.57 ± 1.39	−0.71 ± 1.44	0.14	0.12	0.02 (−0.34, 0.38)	0.01 (−0.37, 0.38)	0.02 (−0.35, 0.39)
12–17.9 mo	−1.56 ± 1.34	−1.68 ± 1.30	−1.20 ± 1.32	−1.37 ± 1.40	0.35*	0.30	0.05 (−0.33, 0.43)	0.04 (−0.32, 0.40)	0.04 (−0.32, 0.40)
18–23.9 mo	−1.93 ± 1.32*	−1.66 ± 1.31	−1.57 ± 1.33	−1.59 ± 1.30	0.37*	0.07	0.30* (0.00, 0.59)	0.30* (0.01, 0.60)	0.30 (−0.01, 0.59)
Underweight, %	24.87	25.66	15.53	15.55	−9.34**	−10.12***	0.77 (−5.59, 7.13)	0.76 (−5.56, 7.08)	0.80 (−5.47, 7.07)
6–11.9 mo	19.10	22.03	11.96	11.31	−7.14*	−10.71**	3.57 (−3.74, 10.88)	3.75 (−3.65, 11.16)	3.76 (−3.60, 11.12)
12–17.9 mo	25.93	29.37	17.07	17.59	−8.86*	−11.78*	2.92 (−7.48, 13.32)	2.82 (−7.01, 12.64)	2.93 (−6.87, 12.73)
18–23.9 mo	31.83	26.53	18.80	19.36	−13.03**	−7.17*	−5.86 (−14.80, 3.07)	−5.73 (−14.41, 2.96)	−5.86 (−14.51, 2.79)
WAZ	−1.21 ± 1.20	−1.25 ± 1.21	−0.95 ± 1.07	−0.96 ± 1.08	0.26**	0.29**	−0.02 (−0.20, 0.15)	−0.02 (−0.20, 0.16)	−0.02 (−0.20, 0.16)
6–11.9 mo	−0.86 ± 1.24	−0.99 ± 1.33	−0.73 ± 1.12	−0.76 ± 1.09	0.13	0.23*	−0.10 (−0.38, 0.18)	−0.11 (−0.40, 0.18)	−0.10 (−0.39, 0.18)
12–17.9 mo	−1.36 ± 1.14	−1.44 ± 1.10	−1.01 ± 1.06	−1.04 ± 1.05	0.35**	0.40**	−0.05 (−0.36, 0.26)	−0.05 (−0.33, 0.23)	−0.06 (−0.33, 0.22)
18–23.9 mo	−1.50 ± 1.10	−1.38 ± 1.10	−1.18 ± 0.95	−1.17 ± 1.04	0.32**	0.21*	0.11 (−0.11, 0.33)	0.11 (−0.11, 0.32)	0.11 (−0.11, 0.32)
Wasting, %	15.96	16.02	8.80	8.57	−7.17*	−7.45**	0.29 (−6.09, 6.66)	0.42 (−5.79, 6.64)	0.60 (−5.54, 6.74)
6–11.9 mo	16.63	18.82	9.76	8.61	−6.88*	−10.22**	3.33 (−3.42, 10.10)	3.47 (−(−3.34, 10.28)	3.59 (−3.28, 10.45)
12–17.9 mo	17.25	14.67	8.17	6.71	−9.08*	−7.96**	−1.12 (−9.07, 6.82)	−1.33 (−8.74, 6.08)	−1.13 (−8.50, 6.24)
18–23.9 mo	13.31	13.78	8.17	10.64	−5.14	−3.14	−2.00 (−10.34, 6.33)	−1.76 (−10.09, 6.58)	−1.55 (−9.99, 6.88)
WHZ	−0.61 ± 1.21	−0.71 ± 1.25	−0.53 ± 1.15	−0.48 ± 1.18	0.17	0.26**	−0.09 (−0.32, 0.14)	−0.09 (−0.32, 0.14)	−0.09 (−0.33, 0.14)
6–11.9 mo	−0.59 ± 1.34	−0.65 ± 1.38	−0.49 ± 1.19	−0.41 ± 1.25	0.11	0.25*	−0.14 (−0.42, 0.14)	−0.15 (−0.43, 0.14)	−0.15 (−0.43, 0.14)
12–17.9 mo	−0.81 ± 1.20	−0.86 ± 1.15	−0.58 ± 1.20	−0.53 ± 1.12	0.24	0.34*	−0.10 (−0.41, 0.21)	−0.10 (−0.39, 0.19)	−0.11 (−0.40, 0.18)
18–23.9 mo	−0.73 ± 1.14	−0.73 ± 1.13	−0.55 ± 1.03	−0.53 ± 1.14	0.18	0.20*	−0.02 (−0.30, 0.27)	−0.03 (−0.32, 0.26)	−0.03 (−0.31, 0.26)

1 Values are percentages or means ± SDs unless otherwise indicated. *, **, ***Significant change from baseline to endline in intensive and nonintensive areas separately, adjusted for clustering effect at woreda level: *P < 0.05, **P < 0.01, ***P < 0.001. DDEs with clustered SEs compare Alive & Thrive intensive and nonintensive areas in 2015 and 2017. DDE, difference-in-difference estimate; HAZ, height-for-age *z* score; ITT, intent-to-treat; pp, percentage point; T, time; WAZ, weight-for-age *z* score; WHZ, weight-for-height *z* score.

2 Accounts for geographic clustering effect at woreda level only.

3 Accounts for geographic clustering effect, child sex, and child age.

4 Accounts for geographic clustering effect, child sex, child age, and variables that are different at baseline and endline (mother's occupation, institutional delivery, and household dietary diversity).

### Intervention exposure

Exposure to various intervention components in the 3 mo preceding the endline survey was twice as high in intensive compared with nonintensive areas, specifically exposure to IYCF messages or IPC delivered by HEWs during home visit (21.6% compared with 11.8%) or at the health post visit (32.3% compared with 17.9%) and HDTLs during home visits (17.8% compared with 4.2%) (**Table 4**). Compared with households in nonintensive areas, significantly more households in the intensive areas were exposed to A&T-supported AG activities, 36.0% heard messages about raising a baby's chicken, and 22.7% heard about growing a “baby's vegetable garden.” Exposure to any CM was significantly higher in intensive areas, ranging from 18.6% to 54.3% depending on CM activity type, compared with exposure in nonintensive areas (3.8–10.7%). Exposure to MM was 35.4% in the intensive group compared with 16% in the nonintensive group. Among the 4 intervention platforms (IPC, AG, CM, and MM), exposure to any intervention platforms was 80.4% and exposure to all 4 platforms was 6.7% in the intensive areas.

**TABLE 4:** Exposure to interventions among mothers with children aged 6–23.9 mo by intervention group at endline^1^

	Endline 2017	
Indicator	Intensive (*n *= 1360), %	Nonintensive (*n *= 1360), %
IPC		
Received IYCF message during HEW home visit in last 3 mo	21.62^##^	11.76
Received IYCF message during health post visit in last 3 mo	32.28^##^	17.94
Received IYCF message during HDTL home visit in last 3 mo	17.79^###^	4.19
AG		
Received message about raising a “baby's chicken”	35.96^###^	11.40
Received message about raising a “baby's vegetable garden”	22.65^###^	5.96
CM		
Attended food demonstration	54.34^###^	9.93
Attended enhanced community conversations	18.60^###^	3.82
Heard a priest sermon about child feeding and fasting	20.81^#^	10.66
MM		
Heard Sebat Mela radio program	35.37^##^	15.96
No. of intervention platforms (range: 0–4; IPC, AG, CM, MM)		
0	17.72	51.69
1	23.75	30.74
2	27.13	12.13
3	22.35	4.26
4	9.04	1.18

1 Values are percentages. ^#, ##, ###^Significant change between Alive & Thrive intensive and nonintensive areas: ^#^*P *< 0.05, ^##^*P *< 0.01, ^###^*P *< 0.001. AG, agricultural activities; CM, community mobilization; HDTL, health development team leader; HEW, health extension worker; IYCF, infant and young child feeding; IPC, interpersonal communication; MM, mass media.

### Dose–response and path analyses

We observed a significant dose–response association between exposure to the number of intervention platforms and improved CF practices, CF knowledge, and stunting and HAZ (**Table 5**). For minimum dietary diversity, exposure to any single intervention was associated with 1.3–2 times higher odds of practice compared with no exposure. The more platforms to which women were exposed, the higher the odds that minimum dietary diversity among children were achieved; exposure to 3 or 4 platforms was associated with 3.2 higher odds (95% CI: 2.2, 4.6) of minimum dietary diversity. The dose–response relation between intervention exposure and minimum meal frequency was weaker than that for minimum dietary diversity. Exposure to several individual interventions was associated with 1.3–1.6 times higher odds of minimum meal frequency compared with no exposure, and exposure to 3 or 4 platforms was associated with 1.9 higher odds (95% CI: 1.4, 2.6) of minimum meal frequency. For stunting, exposure to IPC from HDTL during home visit was significantly associated with 0.6 times lower odds (95% CI: 0.4, 0.9) of stunting. Although a dose–response relation between the number of intervention platforms and stunting was observed, the association was not statistically significant (*P *= 0.09). For HAZ, exposure to information about raising a baby's chicken and to enhanced community conversations was associated with 0.1–0.2 greater HAZ, and exposure to an increasing number of platforms was significantly associated with increased HAZ (β = 0.2; 95% CI: 0.04, 0.44).

**TABLE 5:** Association between exposure to intervention platforms and CF practices, CF knowledge, stunting, or HAZ among children aged 6–23.9 mo^1^

Indicator	Minimum dietary diversity (*n *= 2720)	Minimum meal frequency (*n *= 2720)	CF knowledge (*n *= 2720)	Stunting (*n *= 2720)	HAZ (*n *= 2720)
IPC					
None	Ref	Ref	Ref	Ref	Ref
Received CF message during HEW home visit in last 3 mo	1.48**^2^ (1.14, 1.91)	1.07 (0.84, 1.36)	0.29* (0.04, 0.54)	1.00 (0.75, 1.34)	0.12 (−0.07, 0.31)
Received CF message during health post visit in last 3 mo	1.42*** (1.11, 1.82)	1.41** (1.12, 1.78)	0.44** (0.21, 0.68)	1.24 (0.91, 1.69)	0.00 (−0.20, 0.21)
Received CF message during HDTL home visit in last 3 mo	1.56* (1.05, 2.32)	1.87*** (1.37, 2.54)	0.33* (0.08, 0.57)	0.63* (0.44, 0.91)	−0.03 (−0.23, 0.18)
AG					
None	Ref	Ref	Ref	Ref	Ref
Received message about raising a “baby's chicken”	1.79*** (1.30, 2.47)	1.16 (0.86, 1.57)	0.54** (0.23, 0.85)	0.88 (0.61, 1.26)	0.17 (−0.02, 0.37)
Received message about growing a “baby's vegetable garden”	1.48* (1.01, 2.16)	1.23 (0.84, 1.78)	0.32* (0.06, 0.57)	0.90 (0.62, 1.32)	0.07 (−0.13, 0.27)
CM					
None	Ref	Ref	Ref	Ref	Ref
Attended food demonstration	1.52* (1.09, 2.11)	1.27 (0.98, 1.66)	0.51*** (0.28, 0.75)	0.81 (0.56, 1.15)	0.14 (−0.08, 0.36)
Attended enhanced community conversations	1.82*** (1.30, 2.55)	1.42* (1.03, 1.96)	0.49*** (0.31, 0.68)	0.82 (0.59, 1.15)	0.12 (−0.02, 0.26)
Heard a priest sermon about child feeding and fasting	1.33* (1.06, 1.68)	1.09 (0.75, 1.58)	0.30* (0.07, 0.52)	1.02 (0.73, 1.41)	0.11 (−0.07, 0.29)
MM					
None	Ref	Ref	Ref	Ref	Ref
Heard Sebat Mela radio program	1.57** (1.18, 2.09)	1.44** (1.15, 1.81)	0.55*** (0.39, 0.70)	0.83 (0.62, 1.12)	0.08 (−0.13, 0.29)
No. of intervention platforms (range: 0–4; IPC, AG, CM, MM)					
0	Ref	Ref	Ref	Ref	Ref
1	1.05 (0.74, 1.48)	1.05 (0.90, 1.23)	0.55** (0.30, 0.81)	0.90 (0.71, 1.16)	0.19 (0.05, 0.32)
2	1.82* (1.14, 2.89)	1.69* (1.13, 2.52)	0.88*** (0.59, 1.17)	0.78 (0.54, 1.13)	0.17 (−0.04, 0.39)
3–4	3.15*** (2.18, 4.57)	1.93*** (1.44, 2.57)	1.16*** (0.89, 1.43)	0.77 (0.57, 1.05)	0.24* (0.04, 0.44)

1 Values are ORs (95% CI) for minimum dietary diversity, minimum meal frequency, and stunting; values are βs (95% CI) for CF knowledge and HAZ. ^*, **, ***^Significantly different: *P < 0.05, **P < 0.01, ***P < 0.001. AG, agricultural activities; CF, complementary feeding; CM, community mobilization; HAZ, height-for-age *z* score; HDTL, health development team leader; HEW, health extension worker; IPC, interpersonal communication; MM, mass media; Ref, reference.

2 Models adjusted for child characteristics (age and sex), maternal characteristics (age, education, and occupation), household characteristics (number of children aged <5 y, food security, and socioeconomic status), and clustering at woreda level.

From path analyses (**Supplemental Figure 1**), we observed significant associations between several platforms (AG, CM, and MM) and egg consumption, which was associated with increased minimum dietary diversity. There was a particularly strong association between raising a baby's chicken and egg consumption and minimum dietary diversity and also between exposure to baby's chicken and HAZ indirectly (egg consumption increased minimum dietary diversity, which was associated with increased HAZ).

## Discussion

The intervention package of intensified interpersonal communication, agricultural activities, community mobilization, and mass media had an impact on some CF practices (minimum dietary diversity and minimum acceptable diet) and stunting in comparison with changes observed with the less intensive interventions within a short 2-y period. Although minimum meal frequency increased significantly in the intensive areas, there was no differential impact. In relation to other anthropometric outcomes, we observed significant declines in underweight and wasting and improvements in HAZ, WAZ, and WHZ in both intensive and nonintensive areas over time, but no differential impacts.

Our findings of impacts on minimum dietary diversity were primarily explained by differential improvement in the consumption of vitamin A-rich fruits and vegetables, which was specifically promoted by program messages during IPC and during food demonstrations. In turn, we also observed differential improvements in maternal knowledge about CF, particularly knowledge about foods for enriching a child's porridge (e.g., milk, eggs, carrots, and green leafy vegetables). Regarding other foods specifically promoted by the interventions (i.e., milk and eggs), we did not observe any significant change in dairy consumption, but there was a significant increase in egg consumption in both intensive and nonintensive areas. This may have resulted from spillover of interventions, as observed in the exposure results in nonintensive areas, including nearly 10% exposure to messages about baby's chicken. Furthermore, some communities within 3 nonintensive woredas and in 4 intensive woredas were involved in the United States Agency for International Development's Empowering the New Generation to Improve Nutrition and Economic Opportunities (ENGINE) project (2011–2016), which implemented interventions similar to those of A&T; thus, some spillover was anticipated. Analyses comparing ENGINE and non-ENGINE woredas, however, showed no major differences in outcomes. In relation to other CF practices such as timely introduction of foods and minimum meal frequency, we observed increases in the intensive group and declines in the nonintensive group. Still, most CF practices remained poor at endline, with 24.9% for minimum dietary diversity, 18.2% for minimum acceptable diet, and 4.5% for consumption of iron-rich foods in the intensive areas. Thus, there is a need for continued efforts to improve these practices.

Although 80% of mothers in the intensive group were exposed to at least 1 of the 4 intervention platforms, exposure across the various IYCF-focused interventions was moderate at endline: IPC was 17.8–32.3%, AG was 22.7–36.0%, CM was 18.6–54.3%, and MM was 35.4%. Thus, no single platform achieved high coverage or >60% among the target beneficiaries. The challenge of achieving high reach of health services and interventions into the communities and target households has been documented in other studies in Ethiopia (17, 31, 32). Although health service coverage has improved in recent years, intervention coverage remains a major challenge. For instance, full vaccination among children aged 12–23 mo increased from 24% to 39% and antenatal care coverage increased from 34% to 62% between 2011 and 2016 (2), leaving room for continued improvement. In 2016 and 2017, there was also political unrest, deeply affecting areas of Amhara; in October 2016, the government declared a state of emergency that lasted for 10 mo, which led to disruptions in program implementation during our study period that may have also contributed to lower coverage. The implementing partner Save the Children put focus on supportive supervision of HEWs and HDTLs and monitoring of program activities to ensure success.

Despite relatively moderate exposure to the interventions, we observed positive effects of these interventions on CF practices and linear growth. CF practices were associated positively with exposure to certain single interventions, but the largest significant associations were with exposure to multiple intervention platforms. Although exposure to IPC from HDTL only was significantly associated with lower odds of stunting, we observed a pattern of lower stunting among those exposed to a greater number of intervention platforms; this pattern was significant for HAZ, confirming the dose–response relation between intensity of intervention exposure and linear growth in our study. Furthermore, exposure to message about raising a baby's chicken was associated with several outcomes. Results from the path analyses corroborated this, observed by a strong association between raising a baby's chicken and HAZ indirectly via egg consumption and minimum dietary diversity, lending support for including this nutrition-sensitive AG intervention as part of the multisectoral approach for improving child nutrition.

The most remarkable finding of our study is the large and significant impact on stunting. The differential decline of −5.6 pp in stunting prevalence in children aged 5–23.9 mo in 2 y (−2.8 pp per year) is double the secular trend (−1.2 pp in stunting in children aged <5 y) (2) and slightly greater than impacts from other studies; a recent evaluation of food rations with behavior change communication and strengthening of health service use during the first 1000-day period in Burundi showed an impact of −7.4 pp in stunting in children aged 24–41.9 mo over 4 y (−1.9 pp per year) (33). Stunting is associated with many different factors beyond improved CF practices. From among the covariates measured in our study, we observed significant changes over time in household hygiene, maternal education and occupation, antenatal care visits, and institutional delivery, which improved more in intensive areas compared with nonintensive areas. It is plausible that these favorable conditions as well as unobserved factors (particularly during pregnancy and before 6 mo of age, which were measured in the intensive areas only), in addition to living in the intensive areas, contributed to the significant impact in stunting.

There were some limitations to our study. First, an evaluation using cross-sectional surveys was used rather than tracking individual children over time. This design allowed us to sample households with children who are the target population for interventions and had the potential of being exposed throughout the study period, but we were precluded from linking child-level exposures to outcomes for the same children. Second, given the focus on CF practices among children aged 6–23.9 mo, children <6 mo of age were not included in both arms of the study sample, so we are uncertain of differences in this younger age group between intervention groups. It is possible that changes in the earlier growth period may have contributed to the differential improvements in stunting at 6–23.9 mo. Third, the SDs for our HAZ measurements were large and exceed the WHO cutoff for high risk of measurement error (SD > 1.3), likely due to error in height measurement or child age estimate that was based on recorded/reported birthdate, particularly in the baseline survey and among the youngest subgroup of children aged 6–11.9 mo. Measurement error would have reduced our ability to detect differences. Last, the study location was confined to 3 western zones in Amhara, which were considered to be more food secure than other zones in the region. The level of food insecurity in these areas, however, was still ∼40%, so they may not be markedly different from other communities throughout other regions.

In conclusion, our study contributes to the evidence that delivering social and behavior change interventions using multiple platforms in Ethiopia is feasible and effective. Intensive interventions delivered through multiple platforms achieved improvements in complementary feeding practices and child stunting within a 2-y period. Given the positive short-term impacts on children's nutrition outcomes, evaluation over longer periods may reveal greater impacts on child nutrition. There is a need for continued efforts, however, to expand intervention coverage and to improve complementary feeding practices in Ethiopia. Behavior change interventions aimed at improving child feeding practices through various delivery platforms involving different sectors may be adapted in other contexts in which such platforms exist and poor practices persist without the constraint of extreme food insecurity.

## Supplementary Material

nxz087_Supplemental_Files

## Notes

Funding provided by the Bill & Melinda Gates Foundation with the governments of Canada and Ireland, through Alive & Thrive, managed by FHI 360; additional financial support for the evaluation study was provided by the CGIAR Research Program on Agriculture for Nutrition and Health (A4NH), led by the International Food Policy Research Institute.

Author disclosures: SSK, PHN, YY, YA, MT, ED, EAF, MTR, and PM, no conflicts of interest. YA, MT, and ED were members of the program implementation team that designed and implemented the interventions discussed in this article. They reviewed the manuscript and provided interpretation of the results, but final decisions for content were made by the primary authors from the evaluation team (SSK, PHN, YY, EAF, MTR, and PM).

Supplemental Tables 1–4 and Supplemental Figure 1 are available from the “Supplementary data” link in the online posting of the article and from the same link in the online table of contents at https://academic.oup.com/jn/.

Abbreviations used: AG, agricultural activities; A&T, Alive & Thrive; BF, breastfeeding; CF, complementary feeding; CM, community mobilization; DDE, difference-in-difference estimate; EBF, exclusive breastfeeding; HAZ, height-for-age *z* score; HDTL, health development team leader; HEW, health extension worker; IPC, interpersonal counseling; IYCF, infant and young child feeding; MM, mass media; pp, percentage point; WAZ, weight-for-age *z* score; WHZ, weight-for-height *z* score.
