# Supplementary material for: Behavior Change Interventions Delivered through Interpersonal Communication, Agricultural Activities, Community Mobilization, and Mass Media Increase Complementary Feeding Practices and Reduce Child Stunting in Ethiopia
Source: J Nutr. 2019 Jun 5;149(8):1470–81. doi: 10.1093/jn/nxz087 (PMC6686053; doi:10.1093/jn/nxz087)
Supplement: nxz087_Supplemental_Files [file nxz087_supplemental_files.zip › Online Supporting Materials_Figure1_14March2019.pdf]

Supplementary Data

Supplemental Figure 1. Paths from exposure to intervention platforms to HAZ at endline[1]

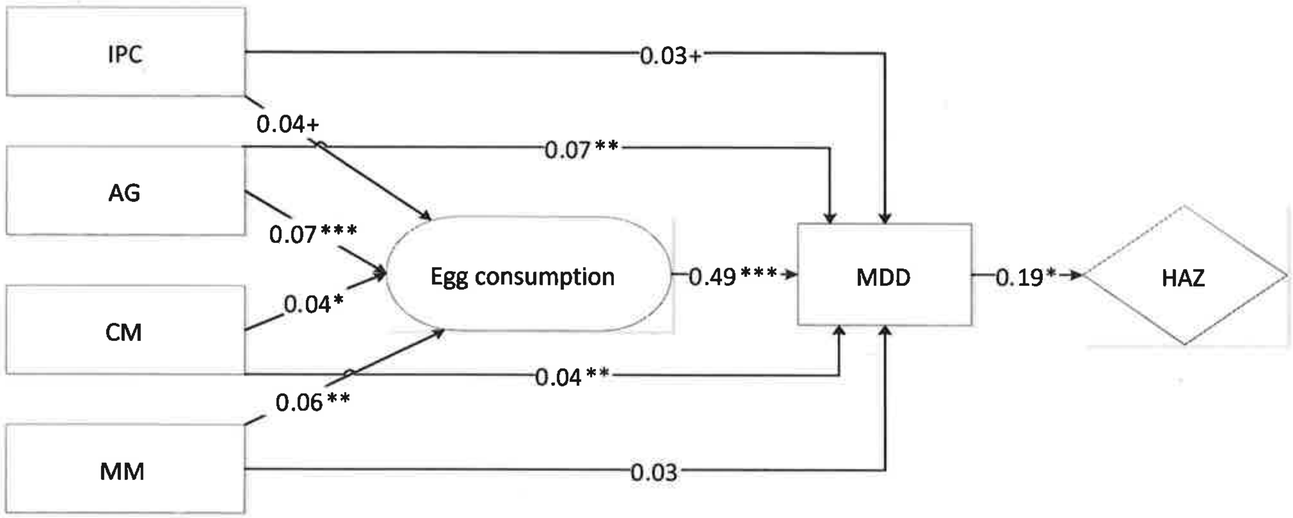

[1]Values are coefficients. +*P*<0.1, * *P* <0.05, ** *P* <0.01, *** *P* <0.001. AG: agricultural activities (specified as raising "baby's chicken" for this model); CM: community mobilization; HAZ: Height-for-age Z-score; IPC: interpersonal communication; MDD: minimum dietary diversity.
