# Supplementary material for: Behavior Change Interventions Delivered through Interpersonal Communication, Agricultural Activities, Community Mobilization, and Mass Media Increase Complementary Feeding Practices and Reduce Child Stunting in Ethiopia
Source: J Nutr. 2019 Jun 5;149(8):1470–81. doi: 10.1093/jn/nxz087 (PMC6686053; doi:10.1093/jn/nxz087)
Supplement: nxz087_Supplemental_Files [file nxz087_supplemental_files.zip › Online Supporting Materials_Table1_27March2019.pdf]

Supplemental Table 1. WHO recommended IYCF indicators[1] by intervention group and survey round[2]

Indicator	Age group (months)	Baseline 2015 Intensive (*n*=1748)	Baseline 2015 Non-intensive (*n*=1318)	Endline 2017 Intensive (*n*=1760)	Endline 2017 Non-intensive (*n*=1360)	Intensive T2-T1 pp	Non-intensive T2-T1 pp	Pure ITT[3] DDE pp (95% CI)	Adjusted ITT[4] DDE pp (95% CI)	Fully adjusted[5] DDE pp (95% CI)
Early initiation of breastfeeding (within 1 h of birth)	6-23.9	38.10	38.85	53.68[#6]	47.28	15.57**[7]	8.43*	7.14 (-3.08, 17.37)	7.14 (-3.08, 17.37)	6.98 (-3.43, 17.40)
Continued breastfeeding at 1 y	12-15.9	98.02	96.56	97.88	96.52	-0.14	-0.04	-0.10 (-3.90, 3.71)	-0.10 (-3.89, 3.69)	0.09 (-3.80, 3.98)
Continued breastfeeding at 2 y	20-23.9	94.74	93.10	93.06	92.89	-1.68	-0.21	-1.46 (-8.75, 5.82)	-1.44 (-8.72, 5.83)	-1.63 (-8.57, 5.30)
Introduction of (semi) solid or soft foods	6-8.9	58.68	56.85	61.30[+]	51.11	2.63	-5.74	8.37 (-4.36, 21.10)	8.22 (-3.59, 20.02)	7.70 (-4.40, 19.80)
Minimum dietary diversity	6-23.9	5.20	4.93	24.85[#]	18.01	19.66***	13.08***	6.57 (-0.92, 14.07)	6.41 (-0.86, 13.69)	6.27 (-0.92, 13.46)
Minimum meal frequency[8]	6-23.9	56.78	53.64	62.50[#]	52.43	5.72	-1.22	6.94 (-4.57, 18.45)	6.46 (-4.45, 17.38)	6.67 (-4.25, 17.58)
Minimum acceptable diet[9]	6-23.9	4.14	2.96	18.24[#]	11.54	14.09***	8.59***	5.51 (-0.44, 11.46)	5.39 (-0.40, 11.17)	5.25 (-0.37, 10.87)
Consumption of iron-rich foods[10]	6-23.9	3.46	3.41	4.49	5.51	1.02	2.10*	-1.08 (-3.56, 1.40)	-1.09 (-3.59, 1.40)	-1.32 (-3.85, 1.22)

[1] Reference (24)
[2] Values are percentages. DDE: difference-in-difference estimate; ITT, intent-to-treat; IYCF: infant and young child feeding; pp: percentage point; T, time; WHO: World Health Organization. DDEs with clustered SEs comparing A&T intensive and non-intensive areas in 2015 and 2017.
[3] Accounts for geographic clustering effect at woreda level only.
[4] Accounts for geographic clustering effect, child sex, and child age.
[5] Accounts for geographic clustering effect, child sex, child age, and variables that are different at baseline and endline (mother's occupation, institutional delivery, and HH dietary diversity).
[6] Significant change between A&T intensive and non-intensive areas in the same survey round, adjusted for clustering effect at woreda level: #*P* <0.05.
[7] Significant change from baseline to endline in intensive and non-intensive areas separately, adjusted for clustering effect at woreda level: **P* <0.05, ***P* <0.01, ****P* <0.001.
[8] Minimum is defined as 2 times for breastfed infants 6-8 months; 3 times for breastfed children 9-23.9 months; 4 times for non-breastfed children 6-23.9 months. "Meals" include both meals and snacks, and frequency is based on mother's report.
[9] Acceptable diet is defined as who had at least the minimum dietary diversity and the minimum meal frequency during the previous day.
[10] Iron-rich or iron-fortified foods include flesh foods, commercially fortified foods especially designed for infants and young children that contain iron, or foods fortified in the home with a micronutrient powder containing iron.
