# Supplementary material for: Behavior Change Interventions Delivered through Interpersonal Communication, Agricultural Activities, Community Mobilization, and Mass Media Increase Complementary Feeding Practices and Reduce Child Stunting in Ethiopia
Source: J Nutr. 2019 Jun 5;149(8):1470–81. doi: 10.1093/jn/nxz087 (PMC6686053; doi:10.1093/jn/nxz087)
Supplement: nxz087_Supplemental_Files [file nxz087_supplemental_files.zip › Online Supporting Materials_Table2_27March2019.pdf]

Supplemental Table 2. Social desirability score among mothers with children 6-23.9 months by intervention group and survey round[1]

Indicator	Baseline 2015 Intensive (*n*=1328) %	Baseline 2015 Non-intensive (*n*=1318) %	Endline 2017 Intensive (*n*=1360) %	Endline 2017 Non-intensive (*n*=1360) %
Give up doing something because you don't think you have the ability (NO=1)	23.0	23.9	21.0	26.3
Feel like not listening to people even if you know they are right (NO=1)	14.2	14.2	16.2	16.0
Irritated/annoyed by people who ask you to do something for them (NO=1)	10.9	11.7	13.8	14.2
Are always courteous, even to people who are disagreeable/not pleasant (YES=1)	59.7	57.9	74.3	69.2
When you make a mistake, you are always willing to admit it (YES=1)	85.8	84.5	84.0	78.2
	**Mean ± SD**	**Mean ± SD**	**Mean ± SD**	**Mean ± SD**
SDS score (range 0-5)	4.0 ± 1.0	3.9 ± 1.0	4.1 ± 1.1	3.9 ± 1.1

[1] Values are percentages or means ± SDs. SDS: social desirability score
