# Supplementary material for: Behavior Change Interventions Delivered through Interpersonal Communication, Agricultural Activities, Community Mobilization, and Mass Media Increase Complementary Feeding Practices and Reduce Child Stunting in Ethiopia
Source: J Nutr. 2019 Jun 5;149(8):1470–81. doi: 10.1093/jn/nxz087 (PMC6686053; doi:10.1093/jn/nxz087)
Supplement: nxz087_Supplemental_Files [file nxz087_supplemental_files.zip › Online Supporting Materials_Table3_27March2019.pdf]

Supplemental Table 3. Association between social desirability score and IYCF indicators by intervention group at endline[1]

	SDS			
**Indicator**	**Low (0-2) %**	**Medium (3) %**	**High (4) %**	**Very high (5) %**
Continued BF at 12 months				
Non-intensive	100.0	100.0	96.0	99.2
Intensive	100.0	100.0	96.4	100.0
Introduction of solid, semi-solid foods				
Non-intensive	39.5	51.7	56.9	51.7
Intensive	63.6	50.0	56.7	68.0
Minimum dietary diversity				
Non-intensive	28.0	32.3	31.5	33.3
Intensive	31.9	34.8	36.3	38.1
Minimum meal frequency				
Non-intensive	54.7	54.8	59.9	62.6
Intensive	66.1	63.5	67.6	68.6
Minimum acceptable diet				
Non-intensive	19.3	18.2	21.8	23.4
Intensive	22.7	25.9	26.5	28.7
Consumption of iron-rich foods				
Non-intensive	4.4	11.2***[2]	3.9	3.6
Intensive	4.2	6.7	5.1	3.3

[1] Values are percentages. BF: breastfeeding; IYCF: infant and young child feeding; SDS: social desirability score.
[2] Significant difference: ****P* <0.001.
