# Supplementary material for: Behavior Change Interventions Delivered through Interpersonal Communication, Agricultural Activities, Community Mobilization, and Mass Media Increase Complementary Feeding Practices and Reduce Child Stunting in Ethiopia
Source: J Nutr. 2019 Jun 5;149(8):1470–81. doi: 10.1093/jn/nxz087 (PMC6686053; doi:10.1093/jn/nxz087)
Supplement: nxz087_Supplemental_Files [file nxz087_supplemental_files.zip › Online Supporting Materials_Table4_27March2019.pdf]

Supplemental Table 4. Complementary feeding knowledge among mothers with children 6-23.9 months by intervention group and survey round[1]

Indicator	Baseline 2017 Intensive (*n*=1328) %	Baseline 2017 Non-intensive (*n*=1318) %	Endline 2017 Intensive (*n*=1360) %	Endline 2017 Non-intensive (*n*=1360) %	DDE[2] pp/mean diff.
Baby should start to receive liquids other than breast milk at 6 mo	78.46	78.30	91.99[##3]	83.01	8.81*[4] (0.22, 17.39)
Baby should start to receive foods in addition to breast milk at 6 mo	76.43	77.69	88.31[#]	80.15	9.42* (1.32, 17.52)
Special foods to complement breast milk are porridge enriched with milk, egg or vegetables	57.61	54.78	92.43[###]	75.00	14.60** (6.74, 22.46)
A 6–9-mo old child should be fed foods other than breast milk 2-3 times a day.	97.14	96.81	98.31	96.84	1.15 (-2.01, 4.30)
A 12–24-mo old child should be fed foods other than breast milk 3-6 times a day.	96.76	95.68	96.62	93.68	1.85 (-3.99, 7.70)
A baby 6-24 mo old should eat animal source food such as eggs, milk or meat everyday	40.74	40.59	53.09	55.74	-2.79 (-14.35, 8.76)
A 1-y old child should not eat alone without any help/assistance	94.88	96.66	93.31	93.75	1.34 (-1.44, 4.12)
A 1-y old child should not eat only the same foods as the rest of the family	90.44	93.47	96.03[##]	92.87	6.20** (1.88, 10.52)
A sick child should be fed the same as usual or more frequently than usual.	70.71	70.18	78.09	73.24	4.33 (-7.06, 15.72)
After illness, feed more food than usual or an extra meal every day for 2 weeks	51.13	56.90	55.74	49.04	12.47 (-0.85, 25.78)
Know at least 4 food groups that children need to grow and develop their brain	22.14	19.50	44.63[#]	35.96	6.04 (-3.53, 15.60)
Know ways to encourage children to eat their foods	94.28	94.92	97.57	97.06	1.15 (-2.23, 4.54)
	**Mean ± SD**	**Mean ± SD**	**Mean ± SD**	**Mean ± SD**	
CF knowledge score (range 0-12)	8.71 ± 1.61	8.75 ± 1.58	9.86 ± 1.47[###]	9.26 ± 1.69	0.65** (0.25, 1.05)

[1] Values are percentages or means ± SDs, unless otherwise indicated. CF: complementary feeding; DDE: difference-in-difference estimate.
[2] DDE between baseline and endline adjusted for clustering effect at woreda level only.
[3] Significant change between A&T intensive and non-intensive areas in the same survey round, adjusted for clustering effect at woreda level: #*P* <0.05, ##*P* <0.01, ###*P* < 0.001.
[4] Significant change from baseline to endline in intensive and non-intensive areas separately, adjusted for clustering effect at woreda level: **P* <0.05, ***P* <0.01.
